# Supplementary material for: Study protocol of validating a numerical model to assess the blood flow in the circle of Willis
Source: BMJ Open. 2020 Jun 4;10(6):e036404. doi: 10.1136/bmjopen-2019-036404 (PMC7279649; doi:10.1136/bmjopen-2019-036404)
Supplement: Author's manuscript [file bmjopen-2019-036404.draft_revisions.pdf]

BMJ Open

BMJ Open is committed to open peer review. As part of this commitment we make the peer review history of every article we publish publicly available.

When an article is published we post the peer reviewers' comments and the authors' responses online. We also post the versions of the paper that were used during peer review. These are the versions that the peer review comments apply to.

The versions of the paper that follow are the versions that were submitted during the peer review process. They are not the versions of record or the final published versions. They should not be cited or distributed as the published version of this manuscript.

BMJ Open is an open access journal and the full, final, typeset and author-corrected version of record of the manuscript is available on our site with no access controls, subscription charges or pay-per-view fees (http://bmjopen.bmj.com).

If you have any questions on BMJ Open's open peer review process please email info.bmjopen@bmj.com

Validation of a numerical model to assess the blood flow in the circle of Willis

Journal:	*BMJ Open*
Manuscript ID	bmjopen-2019-036404
Article Type:	Protocol
Date Submitted by the Author:	13-Dec-2019
Complete List of Authors:	Shen, Yuanyuan; UMCG, Neurosurgery<br>Wei, Yanji; University of Groningen, Engineering and Technology Institute Groningen, Faculty of Science & Engineering,<br>Bokkers, Reinoud; UMCG, Radiology, Medical Imaging Center<br>Uyttenboogaart, Maarten; UMCG, Neurology; UMCG, Radiology, Medical Imaging Center<br>van Dijk, J. Marc C.; UMCG, Neurosurgery
Keywords:	Stroke < NEUROLOGY, NEUROSURGERY, Neuroradiology < RADIOLOGY & IMAGING

SCHOLARONE™
Manuscripts

I, the Submitting Author has the right to grant and does grant on behalf of all authors of the Work (as defined in the below author licence), an exclusive licence and/or a non-exclusive licence for contributions from authors who are: i) UK Crown employees; ii) where BMJ has agreed a CC-BY licence shall apply, and/or iii) in accordance with the terms applicable for US Federal Government officers or employees acting as part of their official duties; on a worldwide, perpetual, irrevocable, royalty-free basis to BMJ Publishing Group Ltd ("BMJ") its licensees and where the relevant Journal is co-owned by BMJ to the co-owners of the Journal, to publish the Work in this journal and any other BMJ products and to exploit all rights, as set out in our licence.

The Submitting Author accepts and understands that any supply made under these terms is made by BMJ to the Submitting Author unless you are acting as an employee on behalf of your employer or a postgraduate student of an affiliated institution which is paying any applicable article publishing charge ("APC") for Open Access articles. Where the Submitting Author wishes to make the Work available on an Open Access basis (and intends to pay the relevant APC), the terms of reuse of such Open Access shall be governed by a Creative Commons licence – details of these licences and which Creative Commons licence will apply to this Work are set out in our licence referred to above.

Other than as permitted in any relevant BMJ Author's Self Archiving Policies, I confirm this Work has not been accepted for publication elsewhere, is not being considered for publication elsewhere and does not duplicate material already published. I confirm all authors consent to publication of this Work and authorise the granting of this licence.

For peer review only

VALIDATION OF A NUMERICAL MODEL TO ASSESS THE BLOOD FLOW IN THE CIRCLE OF WILLIS

YUANYUAN SHEN MD[1], YANJI WEI PHD[2], REINOUD P. H. BOKKERS MD PHD[3], MAARTEN UYTTENBOOGAART MD PHD[3,4], J. MARC C. VAN DIJK MD PHD[1]

AFFILIATIONS

University of Groningen, Groningen, The Netherlands
[1.] Department of Neurosurgery, University Medical Center Groningen,
[2.] Engineering and Technology Institute Groningen, Faculty of Science & Engineering,
[3.] Department of Radiology, Medical Imaging Center, University Medical Center Groningen,
[4.] Department of Neurology, University Medical Center Groningen,

CORRESPONDING AUTHOR:

Yuanyuan Shen, MD
Department of Neurosurgery,
University Medical Center Groningen,
University of Groningen,
Groningen, The Netherlands.
Email: y.shen@umcg.nl

KEY WORDS

Circle of Willis; Numerical model; Cerebrovascular Disorder; Cerebral Blood Flow; Subarachnoid Hemorrhage

WORD COUNT: 2111

For peer review only

ABSTRACT

Introduction We developed a zero-dimensional (0D) model to assess the patient- specific hemodynamics in the circle of Willis (CoW). Similar numerical models for simulating the cerebral blood flow had only been validated qualitatively in healthy volunteers by MRA and TCD. This study aims to validate whether a numerical model can simulate patient- specific blood flow in the CoW under pathological conditions.

Methods and analysis This study is a diagnostic accuracy study. We aim to collect data from a previously performed prospective study that involved patients with aneurysmal subarachnoid hemorrhage (aSAH) receiving both TCD and brain CTA at the same day. The cerebral flow velocities are calculated by the 0D model, based on the vessel diameters measured on the CTA of each patient. In this study, TCD is considered the gold standard for measuring flow velocity in the CoW. The agreement will be analyzed using Pearson correlation coefficients.

Ethics and dissemination This study protocol has been approved by the Medical Ethics Review Board of the University Medical Center Groningen (UMCG): METc2019/103. The results will be submitted to an international scientific journal for peer-reviewed publication.

Trial registration number NL8114

STRENGTHS AND LIMITATIONS OF THIS STUDY

1. This study is the first to check whether a numerical model can reliably simulate patient specific flow velocities in circle of Willis in sub-acute phase of aSAH.
2. The dataset for validation was collected prospectively; both CTA and TCD were done on the same day as a routine clinical procedure by professionals.
3. The accuracy of “gold standard” in this study is highly depends on the operator. The TCD exams within this study were done by different physicians as routines.

For peer review only

INTRODUCTION

Background and rationale

Cerebrovascular disorder (CVD) involves a wide range of vascular disorders that lead to insufficient supply of oxygen and nutrition to the brain. The most severe example is stroke, which has the worst outcome and has become the fifth cause of death in United States in 2017 [1]. Since cerebral blood flow (CBF) is critical during the pathological progress of CVD, the ability to accurately measure CBF is crucial.

Transcranial Doppler (TCD) nowadays has become the most common bedside tool to assess CBF. TCD noninvasively provides real-time cerebral blood flow velocities in proximal cerebral arteries, including its waveform pattern, but its accuracy is highly operator-dependent. Magnetic resonance (MR) sequences can provide flow rate [2] and perfusion territory [3,4] of major cerebral arties. However, MR demands that subjects are immobile for a longer period and has several contra-indications, such as metal implants. Recently, it was reported that dynamic contrast-enhanced near-infrared spectroscopy could assess CBF at the bedside [5], which compensates for the disadvantages of MR, but this technique still needs to be validated.

Many fluid dynamic models have been developed to investigate more information about the CBF or to collect the CBF with relatively less demanding on the patient. These numerical models can simulate blood perfusion from the level of major cerebral arteries [6] to the level of microvascular circulation in the grey and white matter [7]. In addition, it can simulate hemodynamic parameters such as wall shear stress and oscillating shear stress [8–10] , as well as that it can predict the hemodynamic situation after a change in arterial network structure [11,12].

Currently, the application of numerical models is mainly restricted to a laboratory setting, because of its complicated input and simulation, but also due to insufficient validation for use in clinical practice, since many hemodynamic parameters cannot be measured in vivo. The accuracy of simulated flow velocity and pressure has been validated with TCD and MR in young volunteers [13–15] and in geriatric suspected stroke patients [16]. Most of the measurements were validated qualitatively. However, in an acute pathological conditions many physiological characteristics of the blood circulation change, e.g. viscoelasticity of the vessel wall and hydrostatic pressure. Whether a numerical model still can simulate CBF as accurate as under a normal physiological condition remains unknown.

Therefore, we developed a 0D model and aim to use only the CoW vessel diameter as input, in order to simulate CBF within the CoW in patients with aSAH. This validation protocol is a fundamental step for subsequent research on the configuration of CoW and CVD.

Objectives

This study aims to quantitatively validate a 0D model with TCD in aSAH patients.

For peer review only

METHODS

We are to collect the data of aSAH patients who underwent TCD and brain CTA at the same time. Then analysis the agreement between the flow velocity of the CoW simulated by the 0D model and the flow velocity of the CoW measured by TCD. The flowchart of this diagnostic accuracy study is shown in Figure 1.

STUDY POPULATION

For this validation study we aim to collect data from a previously performed prospective study with 59 adult patients diagnosed with aSAH within 4 days after onset [17]. Moribund patients or patients unable to finish CTA or TCD exam were excluded from that study. Paired TCD and CTA were performed twice during the first 2 weeks after aSAH in every participant.

Paired CTA and TCD performed in one patient at the same time will be considered as one investigation. Investigation is to be excluded if the time interval between CTA and TCD exam is longer than 24 hours. Although aSAH patients are prone to experience cerebral vasospasm, we assume that the diameter of the arteries of the CoW remain unchanged between CTA and TCD within one investigation.

CTA image quality is to be checked by two board certified radiologists. In case of insufficient CTA image quality, due to coil/clip artifacts or insufficient timing of CTA , investigation will be excluded.

FLOW VELOCITY MEASUREMENT

TCD was performed by experienced neurophysiology technicians as part of clinical routine. Flow velocities at 11 different locations of 9 arteries of the CoW were reported: (1) distal cervical part of internal carotid artery (ICA); (2) proximal and distal middle cerebral artery (MCA); (3) anterior cerebral artery (ACA); (4) basilar artery (BA); (5) posterior cerebral artery (PCA). Peak systolic velocity (Vs); end diastolic velocity (Vd); and mean velocity (Vm) were recorded in cm/s.

DIAMETER MEASUREMENT

The diameter of each segment of the CoW is to be measured by semi-automatical central lumen calculation on CTA images, using the workstation TeraReconAquariusNET iNtuition Viewer, version 4.4.13.P4. The CTA was performed with iodine contrast agent (Iomeron).

The diameter of each segment of the CoW is to be measured at two points: start and end. The extracranial ICA point is also measured. The start point and end point of each segment are defined as below:

1. ACom (anterior communicating artery): between left and right ACA. One point in the middle, the other point after the junction.
2. ACA: start after bifurcation of ICA to MCA and ACA, middle at before ACom starts, end after junction of ACom.
3. MCA: start after bifurcation of ICA to MCA and ACA; end before bifurcation.
4. ICA: start at the entrance of the petrous bone (end of C1 part), middle at Ophthalmic artery (C6 part); end before bifurcation to MCA and ACA (C7 part).
5. PCom (posterior communication artery): between ipsilateral ICA and PCA. One point in the middle, the other point after the junction.
6. PCA: start after BA bifurcation to left and right PCA; middle at where it meets PCom, end after the junction.

For peer review only

7. BA: start at the vertebrobasilar junction; end before bifurcation to left and right PCA.

The measuring points for diameters from CTA and for flow velocities from TCD are described in Figure 2.

Flow velocity simulation

Hemodynamic model

The hemodynamics in the CoW are described using a lumped parameter model, the 0D model. This model assumes that blood is an incompressible fluid, and that arteries are thin, homogeneous deformable tubes behaving in a viscoelastic manner with a linear Kelvin–Voigt model [18]. The friction losses were calculated with a flow regime (laminar/ turbulent) dependent friction factor using the Haaland approximation. The local losses are neglected in the present model, since accounting such loss in the simulations did not have a significant effect on pressure or flow waveforms [19].

Different from conventional 0D models that applied the hydraulic-electrical analogue, the present model is based on Simulink[TM], using Simscape Fluids library, which can provide an intuitive and easy way to model the blood circulation system as the hydraulic systems within a block diagram environment (Figure 3). This allows us to easily customize the connectivity of the artery network. Each segment in the arterial network is described with "Segmented Pipeline" block, which accounts its resistive, fluid inertia, and wall compliance.

Boundary conditions and initialization

At the root of the ascending aorta, a periodical flow rate that describes the systole with a half sinusoidal wave and the diastole with zero flow rate. At the end of each terminal branch, the present model adopts three-element Windkessel model (WK3) as the downstream condition, which consists of two resistances and a compliance [13] in order to minimize the non-physiological reflection. The WK3 is connected with a constant pressure of 5 mmHg which represented the pressure at the entrance of the venous system.

The model is initialized with flow rate $Q = 0$ and pressure $P = 0$ in all the segments. All simulations are allowed to run for ten cardiac cycles to achieve steady-periodic solution in the present study. The results of the last cycle are to be selected for analysis.

Physiological data

The basic model in this study consists of an arterial network with 33 segments. The arterial network provides a detailed description of the arterial circulation, particularly the cerebral circulation (**Error! Reference source not found.**). The geometrical parameters of an artery include length and diameter. The diameters of the arteries in the CoW are to be measured on the CTA images, while other studies collected diameters based on data from the literature [15]. Since the length of the cerebral arteries (~ 10^{-2} m) is much shorter than the wavelength of the pulse waves (~10^{0} m), and measuring length on medical imaging is not straightforward, makes that length is less practical to be used as a diagnostic parameter than diameter. It is, therefore, decide to only use diameters to represent the patient specific CoW in the simulations in order to the get patient specific cerebral hemodynamics. The length remains the same as that of Alastruey et al [13].

The viscoelastic coefficients of the arteries are difficult to be estimated. Ghigo et al [18] investigated the viscoelasticity of the arteries on animals using the Kelvin–Voigt model. They found that the viscoelastic relaxation time (the ratio between the viscoelastic coefficient and the Young's modulus) are nearly

For peer review only

constant throughout the network. Due to lacking of in-vivo human measurements, the same constant viscoelastic relaxation time as that in Ghigo et al (0.02 s) is to be used in the whole network.

Data analysis

Statistical analysis

All data is to be analyzed with Statistical Package of Social Sciences (SPSS). Lin's Concordance Correlation Coefficient is to be tested first to check the agreement between TCD and the 0D model. If low agreement shows, Pearson's correlation coefficient is to be tested next if the data is normally distributed, the Spearman's rank correlation coefficient is to be tested if data is not normally distributed. Bland -Altman plots are generated to show the agreement between TCD and the simulation by the 0D model.

Statistical power

There are 118 investigations according to the study design of the previous study before screening. With that sample size, the 95% confidence interval of an expected correlation at 0.8 is 0.73- 0.86; 95% CI of expected r at 0.6 is 0.47-0.70 . If 30% investigations are excluded after screening, the 95% CI of expected r at 0.8 is 0.71-0.87.

ETHICS AND DISSEMINATION

This study protocol has been judged as non-WMO study by the Medical Ethics Review Board of the University Medical Center Groningen (METc UMCG) on February 12th, 2019(METc2019/103). Therefore, no patient consent is needed. The previous study TACTICS has been approved by the METc UMCG(METc2013/051), and was registered in the Dutch trial register: NTR4157.The current study is registered in the Netherlands Trial Register: NL8114.

PROVENANCE AND PEER REVIEW

Not commissioned; externally peer reviewed.

AUTHOR CONTRIBUTIONS

YS as a corresponding researcher, is responsible for the quality of data collection, data analysis, and drafting the manuscript. YW developed the numerical model and performed the simulation. MU provided the patients' data, technically assisted the collection of the data, and critically revised the manuscript. RPB critically revised the manuscript. JMCvD supervised the study and critically revised the manuscript.

FUNDING STATEMENT

YS receives financial support from China Scholarship Council (CSC, File No. 201706320024).

For peer review only

COMPETING INTEREST STATEMENT

The authors report no conflict of interest concerning the methods used in this study or the findings specified in this paper.

REFERENCES

1 Kochanek KD, Xu J, Murphy SL;, *et al.* National Vital Statistics Reports Deaths 2017. 2017;**68**.

2 Amin-Hanjani S, Du X, Pandey DK, *et al.* Effect of age and vascular anatomy on blood flow in major cerebral vessels. *J Cereb Blood Flow Metab* 2015;**35**:312–8.

3 Van Laar PJ, Hendrikse J, Golay X, *et al.* In vivo flow territory mapping of major brain feeding arteries. *Neuroimage* 2006;**29**:136–44.

4 Hartkamp NS, Petersen ET, Chappell MA, *et al.* Relationship between haemodynamic impairment and collateral blood flow in carotid artery disease. *J Cereb Blood Flow Metab* 2018;**38**:2021–32.

5 Milej D, He L, Abdalmalak A, *et al.* Quantification of cerebral blood flow in adults by contrast-enhanced near-infrared spectroscopy: Validation against MRI. *J Cereb Blood Flow Metab* 2019;:0271678X19872564.

6 Liang F, Fukasaku K, Liu H TS. A computational model study of the influence of the anatomy of the circle of willis on cerebral hyperperfusion following carotid artery surgery. *Biomed Eng Online* 2011;**10**:84.

7 Guo L, Li Z, Lyu J, *et al.* On the Validation of a Multiple-Network Poroelastic Model Using Arterial Spin Labeling MRI Data. *Front Comput Neurosci* 2019;**13**:60.

8 Alnaes MS, Isaksen J, Mardal K-A, *et al.* Computation of hemodynamics in the circle of Willis. *Stroke* 2007;**38**:2500–5.

9 Moore S. Computational 3D Modelling of Hemodynamics in the Circle of Willis. 2007.

10 Tanweer O, Wilson TA, Metaxa E, *et al.* A comparative review of the hemodynamics and pathogenesis of cerebral and abdominal aortic aneurysms: lessons to learn from each other. *J Cerebrovasc Endovasc Neurosurg* 2014;**16**:335–49.

11 Khodaee F, Vahidi B, Fatouraee N. Analysis of mechanical parameters on the thromboembolism using a patient-specific computational model. *Biomech Model Mechanobiol* 2016;**15**:1295–305.

12 Neidlin M, Büsen M, Brockmann C, *et al.* A numerical framework to investigate hemodynamics during endovascular mechanical recanalization in acute stroke. *Int j numer method biomed eng* 2016;**32**:e02748.

13 Alastruey J, Parker KH, Peiró J, *et al.* Modelling the circle of Willis to assess the effects of anatomical variations and occlusions on cerebral flows. *J Biomech* 2007;**40**:1794–805.

14 Groen D, Richardson RA, Coy R, *et al.* Validation of patient-specific cerebral blood flow simulation using transcranial doppler measurements. *Front Physiol* 2018;**9**:1–13.

15 Reymond P, Merenda F, Perren F, *et al.* Validation of a One-Dimensional Model of the Systemic Arterial Tree. *Am J Physiol - Hear Circ Physiol* 2009;**297**:208–22.

16 Liu B, Li Q, Wang J, *et al.* A Highly Similar Mathematical Model for Cerebral Blood Flow Velocity in Geriatric Patients with Suspected Cerebrovascular Disease. *Sci Rep* 2015;**5**:1–7.

17 Harst V Der, Joep J, J JW. Transcranial Doppler Versus CT- Angiography for Detection of Cerebral Vasospasm in Relation to Delayed Cerebral Ischemia After Aneurysmal Subarachnoid Hemorrhage A Prospective Single-Center Cohort Study The Transcranial doppler and CT-angiography for Inve. 2019;**44**:1–6.

For peer review only

18 Ghigo A, Wang X-F, Armentano R, *et al.* Linear and nonlinear viscoelastic arterial wall models: application on animals. *J Biomech Eng* 2017;**139**:011003.

19 Matthys KS, Alastruey J, Peiró J, *et al.* Pulse wave propagation in a model human arterial network: Assessment of 1-D numerical simulations against in vitro measurements. *J Biomech* 2007;**40**:3476–86.

For peer review only

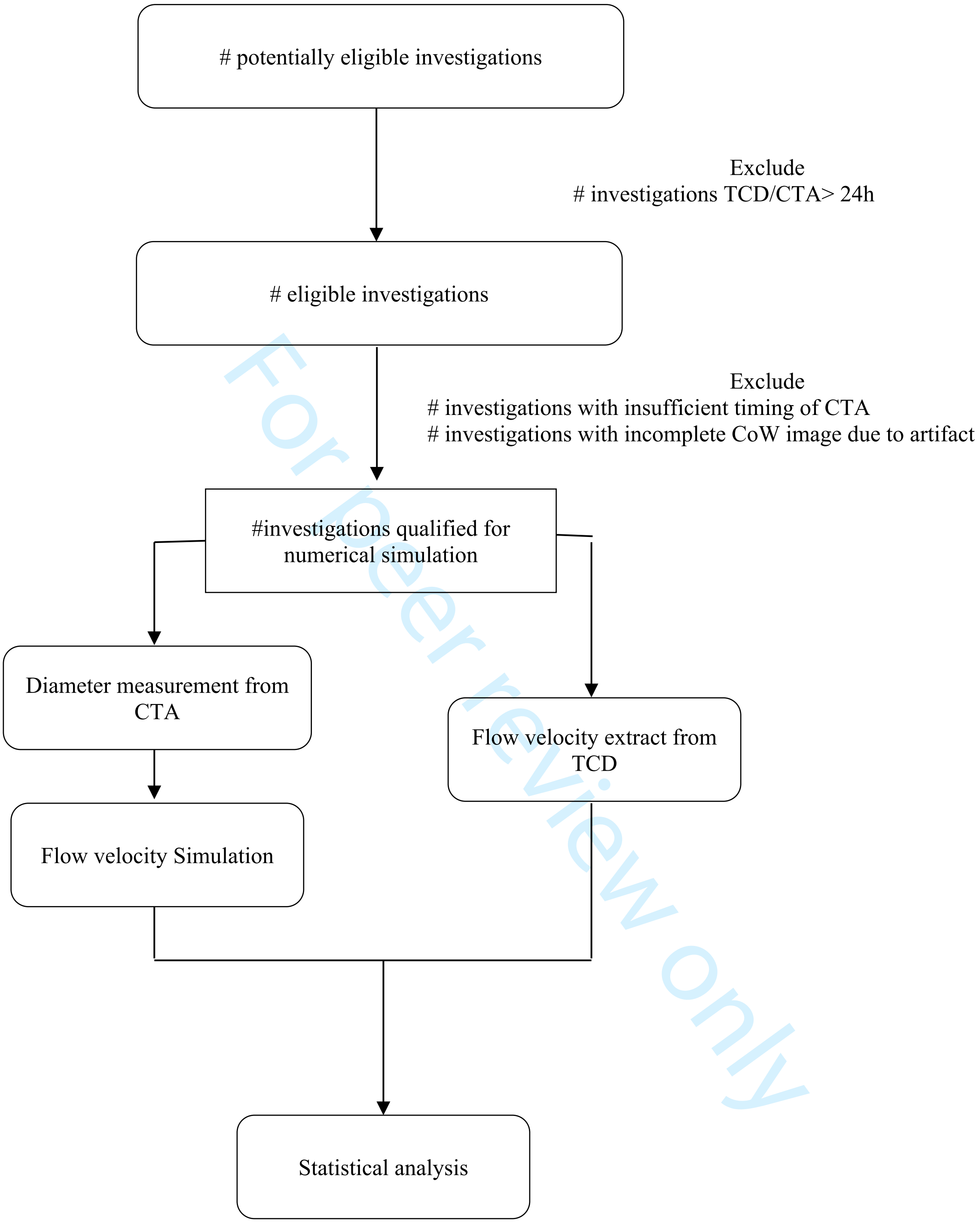

Figure 1. Study flowchart

For peer review only

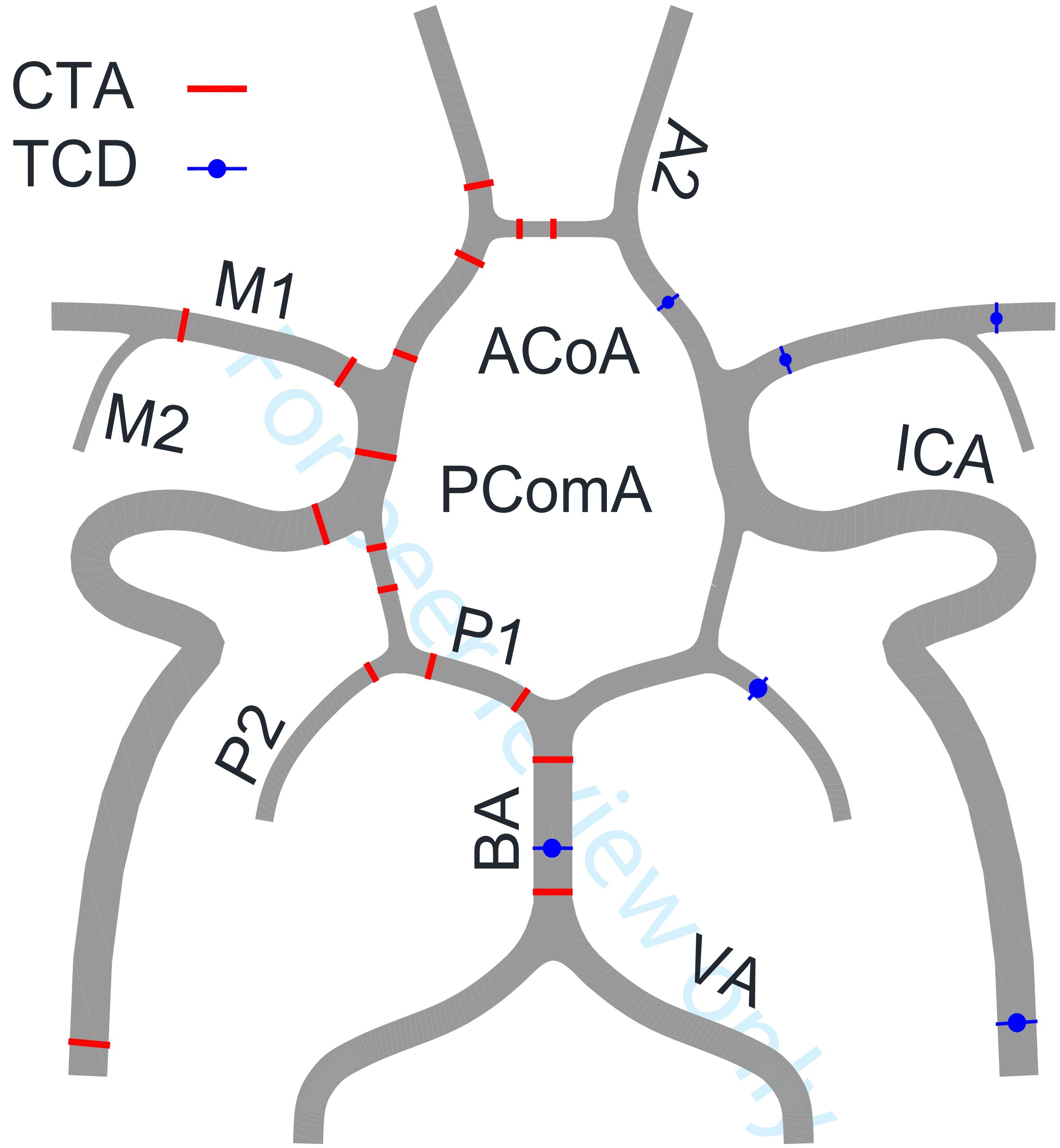

Figure 2. Schematic diagram of measuring point of diameter from CTA and flow velocity from TCD

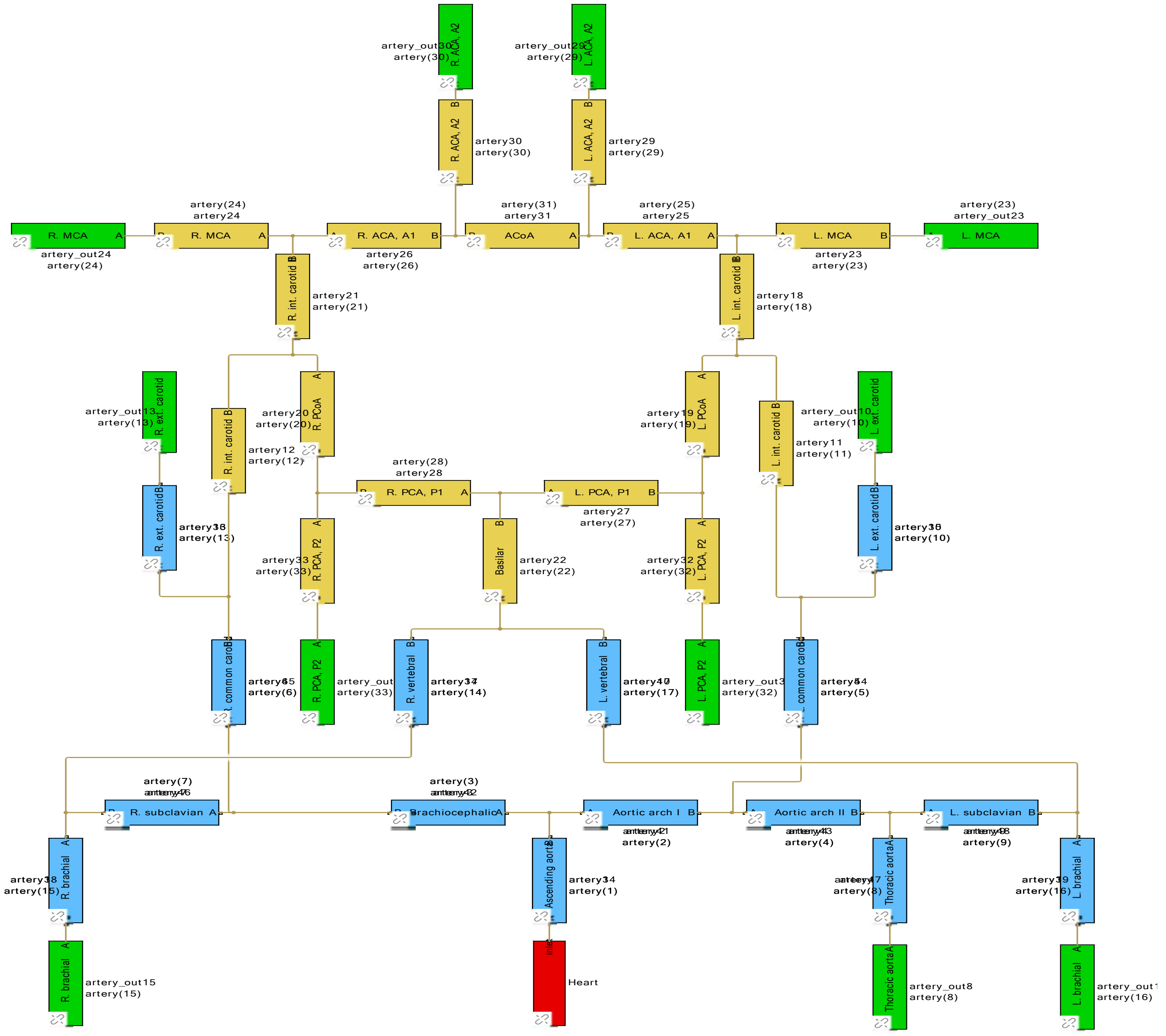

Figure 3. SIMSCAPE™ block diagrams of the arterial network with 33 segments, based on Alastruey et al. [1].

1 Alastruey J, Parker KH, Peiró J, *et al.* Modelling the circle of Willis to assess the effects of anatomical variations and occlusions on cerebral flows. *J Biomech* 2007;**40**:1794–805.

BMJ Open

Study protocol of validating a numerical model to assess the blood flow in the circle of Willis

Journal:	*BMJ Open*
Manuscript ID	bmjopen-2019-036404.R1
Article Type:	Protocol
Date Submitted by the Author:	12-Feb-2020
Complete List of Authors:	Shen, Yuanyuan; UMCG, Neurosurgery<br>Wei, Yanji; University of Groningen, Engineering and Technology Institute Groningen, Faculty of Science & Engineering,<br>Bokkers, Reinoud; UMCG, Radiology, Medical Imaging Center<br>Uyttenboogaart, Maarten; UMCG, Neurology; UMCG, Radiology, Medical Imaging Center<br>van Dijk, J. Marc C.; UMCG, Neurosurgery
**Primary Subject Heading**:	Neurology
Secondary Subject Heading:	Radiology and imaging
Keywords:	Stroke < NEUROLOGY, NEUROSURGERY, Neuroradiology < RADIOLOGY & IMAGING

SCHOLARONE™ Manuscripts

I, the Submitting Author has the right to grant and does grant on behalf of all authors of the Work (as defined in the below author licence), an exclusive licence and/or a non-exclusive licence for contributions from authors who are: i) UK Crown employees; ii) where BMJ has agreed a CC-BY licence shall apply, and/or iii) in accordance with the terms applicable for US Federal Government officers or employees acting as part of their official duties; on a worldwide, perpetual, irrevocable, royalty-free basis to BMJ Publishing Group Ltd ("BMJ") its licensees and where the relevant Journal is co-owned by BMJ to the co-owners of the Journal, to publish the Work in this journal and any other BMJ products and to exploit all rights, as set out in our licence.

The Submitting Author accepts and understands that any supply made under these terms is made by BMJ to the Submitting Author unless you are acting as an employee on behalf of your employer or a postgraduate student of an affiliated institution which is paying any applicable article publishing charge ("APC") for Open Access articles. Where the Submitting Author wishes to make the Work available on an Open Access basis (and intends to pay the relevant APC), the terms of reuse of such Open Access shall be governed by a Creative Commons licence – details of these licences and which Creative Commons licence will apply to this Work are set out in our licence referred to above.

Other than as permitted in any relevant BMJ Author's Self Archiving Policies, I confirm this Work has not been accepted for publication elsewhere, is not being considered for publication elsewhere and does not duplicate material already published. I confirm all authors consent to publication of this Work and authorise the granting of this licence.

For peer review only

STUDY PROTOCOL OF VALIDATING A NUMERICAL MODEL TO ASSESS THE BLOOD FLOW IN THE CIRCLE OF WILLIS

YUANYUAN SHEN MD[1], YANJI WEI PHD[2], REINOUD P. H. BOKKERS MD PHD[3], MAARTEN UYTTENBOOGAART MD PHD[3,4], J. MARC C. VAN DIJK MD PHD[1]

AFFILIATIONS

University of Groningen, Groningen, The Netherlands
[1.] Department of Neurosurgery, University Medical Center Groningen,
[2.] Engineering and Technology Institute Groningen, Faculty of Science & Engineering,
[3.] Department of Radiology, Medical Imaging Center, University Medical Center Groningen,
[4.] Department of Neurology, University Medical Center Groningen,

CORRESPONDING AUTHOR:

Yuanyuan Shen, MD
Department of Neurosurgery,
University Medical Center Groningen,
University of Groningen,
Groningen, The Netherlands.
Email: y.shen@umcg.nl

KEY WORDS

Circle of Willis; Numerical model; Cerebrovascular Disorder; Cerebral Blood Flow; Subarachnoid Hemorrhage

WORD COUNT: 2236

For peer review only

ABSTRACT

Introduction We developed a zero-dimensional (0D) model to assess the patient- specific hemodynamics in the circle of Willis (CoW). Similar numerical models for simulating the cerebral blood flow had only been validated qualitatively in healthy volunteers by Magnetic Resonance (MR) Angiography and Transcranial Doppler (TCD). This study aims to validate whether a numerical model can simulate patient-specific blood flow in the CoW under pathological conditions.

Methods and analysis This study is a diagnostic accuracy study. We aim to collect data from a previously performed prospective study that involved patients with aneurysmal subarachnoid hemorrhage (aSAH) receiving both TCD and brain Computed Tomography (CT) Angiography at the same day. The cerebral flow velocities are calculated by the 0D model, based on the vessel diameters measured on the CTA of each patient. In this study, TCD is considered the gold standard for measuring flow velocity in the CoW. The agreement will be analyzed using Pearson correlation coefficients.

Ethics and dissemination This study protocol has been approved by the Medical Ethics Review Board of the University Medical Center Groningen (UMCG): METc2019/103. The results will be submitted to an international scientific journal for peer-reviewed publication.

Trial registration number NL8114

STRENGTHS AND LIMITATIONS OF THIS STUDY

1. This is the first study that intends to validate a lumped hemodynamic model in CoW in patients with aSAH.
2. The to-be-validated model is applicable for clinicians.
3. The study can help to understand the cerebral circulation in patients with aSAH.
4. The dataset for validation was collected prospectively as a routine clinical procedure.
5. The accuracy of TCD itself is highly depends on the operator, which limits its accuracy.

For peer review only

INTRODUCTION

BACKGROUND AND RATIONALE

Cerebrovascular disorder (CVD) involves a wide range of vascular disorders that lead to insufficient supply of oxygen and nutrition to the brain. The most severe example is stroke, which has the worst outcome and has become the fifth cause of death in United States in 2017 [1]. Since cerebral blood flow (CBF) is critical during the pathological progress of CVD, the ability to accurately measure CBF is crucial.

TCD nowadays has become the most common bedside tool to assess CBF. TCD noninvasively provides real-time cerebral blood flow velocities in proximal cerebral arteries, including its waveform pattern, but its accuracy is highly operator-dependent. MR sequences can provide flow rate [2] and perfusion territory [3,4] of major cerebral arties. However, MR demands that subjects are immobile for a longer period and has several contra-indications, such as metal implants. Recently, it was reported that dynamic contrast-enhanced near-infrared spectroscopy could assess CBF at the bedside [5], which compensates for the disadvantages of MR, but this technique still needs to be validated.

Many fluid dynamic models have been developed to investigate more information about the CBF or to collect the CBF with relatively less demanding on the patient. Cerebral perfusion can be simulated from the level of major cerebral arteries by lumped model [6] to the level of microvascular circulation in the grey and white matter by multiple-network pro-elastic models [7]. In addition, three dimensional models can simulate hemodynamic parameters such as wall shear stress and oscillating shear stress [8–10] , as well as that it can predict the hemodynamic situation after a change in arterial network structure [11,12].

Currently, the application of numerical models is mainly restricted to a laboratory setting, because of its complicated input and simulation, but also due to insufficient validation for use in clinical practice, since many hemodynamic parameters cannot be measured in vivo. The accuracy of simulated flow velocity and pressure has been validated with TCD and MR in young volunteers [13–15] and in geriatric suspected stroke patients [16]. Most of the measurements were validated qualitatively. However, in an acute pathological condition many physiological characteristics of the blood circulation change, e.g. viscoelasticity of the vessel wall and hydrostatic pressure. Whether a numerical model still can simulate CBF as accurate as under a normal physiological condition remains unknown.

Therefore, we developed a 0D model and aim to use only the CoW vessel diameter as input, in order to simulate CBF within the CoW in patients with aSAH. This validation protocol is a fundamental step for subsequent research on the configuration of CoW and CVD.

OBJECTIVES

This study aims to quantitatively validate a 0D model with TCD in aSAH patients.

For peer review only

METHODS

We are to collect the data of aSAH patients who underwent TCD and brain CTA at the same time. Then analysis the agreement between the flow velocity of the CoW simulated by the 0D model and the flow velocity of the CoW measured by TCD. The flowchart of this diagnostic accuracy study is shown in Figure 1.

Study population

For this validation study we aim to collect data from a previously performed prospective study with 59 adult patients diagnosed with aSAH within 4 days after onset [17]. Moribund patients or patients unable to finish CTA or TCD exam were excluded from that study. Paired TCD and CTA were performed twice during the first 2 weeks after aSAH in every participant.

Paired CTA and TCD performed in one patient at the same time will be considered as one investigation. Investigation is to be excluded if the time interval between CTA and TCD exam is longer than 24 hours. Although aSAH patients are prone to experience cerebral vasospasm, we assume that the diameter of the arteries of the CoW remain unchanged between CTA and TCD within one investigation.

CTA image quality is to be checked by two board certified radiologists. In case of insufficient CTA image quality, due to coil/clip artifacts or insufficient timing of CTA, investigation will be excluded.

Flow velocity measurement

TCD was performed by experienced neurophysiology technicians as part of clinical routine. Flow velocities at 11 different locations of 9 arteries of the CoW were reported: (1) distal cervical part of internal carotid artery (ICA); (2) proximal and distal middle cerebral artery (MCA); (3) anterior cerebral artery (ACA); (4) basilar artery (BA); (5) posterior cerebral artery (PCA). Peak systolic velocity (Vs); end diastolic velocity (Vd); and mean velocity (Vm) were recorded in cm/s.

Diameter measurement

The diameter of each segment of the CoW is to be measured by semi-automatical central lumen calculation on CTA images, using the workstation TeraReconAquariusNET iNtuition Viewer, version 4.4.13.P4. The CTA was performed with iodine contrast agent (Iomeron).

The diameter of each segment of the CoW is to be measured at two points: start and end. The extracranial ICA point is also measured. The start point and end point of each segment are defined as below:

1. ACom (anterior communicating artery): between left and right ACA. One point in the middle, the other point after the junction.
2. ACA: start after bifurcation of ICA to MCA and ACA, middle at before ACom starts, end after junction of ACom.
3. MCA: start after bifurcation of ICA to MCA and ACA; end before bifurcation.
4. ICA: start at the entrance of the petrous bone (end of C1 part), middle at Ophthalmic artery (C6 part); end before bifurcation to MCA and ACA (C7 part).
5. PCom (posterior communication artery): between ipsilateral ICA and PCA. One point in the middle, the other point after the junction.
6. PCA: start after BA bifurcation to left and right PCA; middle at where it meets PCom, end after the junction.

For peer review only

7. BA: start at the vertebrobasilar junction; end before bifurcation to left and right PCA.

The measuring points for diameters from CTA and for flow velocities from TCD are described in Figure 2.

Flow velocity simulation

Hemodynamic model

The hemodynamics in the CoW are described using a lumped parameter model, the 0D model. This model assumes that blood is an incompressible fluid, and that arteries are thin, homogeneous deformable tubes behaving in a viscoelastic manner with a linear Kelvin–Voigt model [18]. The friction losses were calculated with a flow regime (laminar/ turbulent) dependent friction factor using the Haaland approximation. The local losses are neglected in the present model, since accounting such loss in the simulations did not have a significant effect on pressure or flow waveforms [19].

Different from conventional 0D models that applied the hydraulic-electrical analogue, the present model is based on Simulink[TM], using Simscape Fluids library, which can provide an intuitive and easy way to model the blood circulation system as the hydraulic systems within a block diagram environment (Figure 3). This allows us to easily customize the connectivity of the artery network. Each segment in the arterial network is described with "Segmented Pipeline" block, which accounts its resistive, fluid inertia, and wall compliance.

Boundary conditions and initialization

At the root of the ascending aorta, a periodical flow rate that describes the systole with a half sinusoidal wave and the diastole with zero flow rate. At the end of each terminal branch, the present model adopts three-element Windkessel model (WK3) as the downstream condition, which consists of two resistances and a compliance [13] in order to minimize the non-physiological reflection. The WK3 is connected with a constant outlet pressure which represented the pressure at the entrance of the venous system. Sensitivity analysis showed that the results are insensitivity to the outlet pressure thus a constant pressure of 5 mmHg is used in the simulation.

The model is initialized with flow rate $Q = 0$ and pressure $P = 0$ in all the segments.

Physiological data

The basic model in this study consists of an arterial network with 33 segments. The arterial network provides a detailed description of the arterial circulation, particularly the cerebral circulation (Figure 2). The geometrical parameters of an artery include length and diameter. The diameter of each segment in the CoW is calculated based on equivalent circular truncated cone volume, using the diameters measured at two ends of the artery from CTA images, while other studies collected diameters based on data from the literature [15]. Since the length of the cerebral arteries (~10^{-2} m) is much shorter than the wavelength of the pulse waves (~10^{0} m), and measuring length on medical imaging is not straightforward, makes that length is less practical to be used as a diagnostic parameter than diameter. It is, therefore, decide to only use diameters to represent the patient specific CoW in the simulations in order to the get patient specific cerebral hemodynamics. The length remains the same as that of Alastruey et al [13].

The viscoelastic coefficients of the arteries are difficult to be estimated. Ghigo et al [18] investigated the viscoelasticity of the arteries on animals using the Kelvin–Voigt model. They found that the viscoelastic

For peer review only

relaxation time (the ratio between the viscoelastic coefficient and the Young's modulus) are nearly constant throughout the network. Due to lacking of in-vivo human measurements, the same constant viscoelastic relaxation time as that in Ghigo et al (0.02 s) is to be used in the whole network.

For peer review only

Data analysis

Data collection

For each patient, simulation will run for ten cardiac cycles to achieve steady-periodic state. The results of the last cycle are to be selected for analysis. The output of the model includes the time series of flow rate and pressure at each segment. The derived mean flow velocity at 9 arteries with TCD measurement are collected for statistical analysis.

Statistical analysis

All data is to be analyzed with Statistical Package of Social Sciences (SPSS). Lin's Concordance Correlation Coefficient is to be tested first to check the agreement between TCD and the 0D model. If low agreement shows, Pearson's correlation coefficient is to be tested next if the data is normally distributed, the Spearman's rank correlation coefficient is to be tested if data is not normally distributed. Bland -Altman plots are generated to show the agreement between TCD and the simulation by the 0D model.

Statistical power

There are 118 investigations according to the study design of the previous study before screening. With that sample size, the 95% confidence interval of an expected correlation at 0.8 is 0.73- 0.86; 95% CI of expected r at 0.6 is 0.47-0.70. If 30% investigations are excluded after screening, the 95% CI of expected r at 0.8 is 0.71-0.87.

Patient and Public Involvement

As mentioned above, the data will be collected from a completed prospective study. The objection register will be checked, and the data from those who objected will be excluded from this study. Thus, no informed consent is needed which has been confirmed by local Medical Ethics Review Board. Patients and public are not involved in the study design.

For peer review only

ETHICS AND DISSEMINATION

This study protocol has been judged as non-WMO study by the Medical Ethics Review Board of the University Medical Center Groningen (METc UMCG) on February 12th, 2019(METc2019/103). Therefore, no patient consent is needed. The previous study TACTICS has been approved by the METc UMCG(METc2013/051), and was registered in the Dutch trial register: NTR4157.The current study is registered in the Netherlands Trial Register: NL8114.

PROVENANCE AND PEER REVIEW

Not commissioned; externally peer reviewed.

AUTHOR CONTRIBUTIONS

YS as a corresponding researcher, is responsible for the quality of data collection, data analysis, and drafting the manuscript. YW developed the numerical model and performed the simulation. MU provided

the patients' data, technically assisted the collection of the data, and critically revised the manuscript. RPB critically revised the manuscript. JMCvD supervised the study and critically revised the manuscript.

FUNDING STATEMENT

YS receives financial support from China Scholarship Council (CSC, File No. 201706320024).

COMPETING INTEREST STATEMENT

The authors report no conflict of interest concerning the methods used in this study or the findings specified in this paper.

REFERENCES

1 Kochanek KD, Xu J, Murphy SL;, *et al.* National Vital Statistics Reports Deaths 2017. 2017;**68**.

2 Amin-Hanjani S, Du X, Pandey DK, *et al.* Effect of age and vascular anatomy on blood flow in major cerebral vessels. *J Cereb Blood Flow Metab* 2015;**35**:312–8.

3 Van Laar PJ, Hendrikse J, Golay X, *et al.* In vivo flow territory mapping of major brain feeding arteries. *Neuroimage* 2006;**29**:136–44.

4 Hartkamp NS, Petersen ET, Chappell MA, *et al.* Relationship between haemodynamic impairment and collateral blood flow in carotid artery disease. *J Cereb Blood Flow Metab* 2018;**38**:2021–32.

5 Milej D, He L, Abdalmalak A, *et al.* Quantification of cerebral blood flow in adults by contrast-enhanced near-infrared spectroscopy: Validation against MRI. *J Cereb Blood Flow Metab* 2019;:0271678X19872564.

6 Liang F, Fukasaku K, Liu H TS. A computational model study of the influence of the anatomy of the circle of willis on cerebral hyperperfusion following carotid artery surgery. *Biomed Eng Online* 2011;**10**:84.

7 Guo L, Li Z, Lyu J, *et al.* On the Validation of a Multiple-Network Poroelastic Model Using Arterial Spin Labeling MRI Data. *Front Comput Neurosci* 2019;**13**:60.

8 Alnaes MS, Isaksen J, Mardal K-A, *et al.* Computation of hemodynamics in the circle of Willis. *Stroke* 2007;**38**:2500–5.

9 Moore S. Computational 3D Modelling of Hemodynamics in the Circle of Willis. 2007.

10 Tanweer O, Wilson TA, Metaxa E, *et al.* A comparative review of the hemodynamics and pathogenesis of cerebral and abdominal aortic aneurysms: lessons to learn from each other. *J Cerebrovasc Endovasc Neurosurg* 2014;**16**:335–49.

11 Khodaee F, Vahidi B, Fatouraee N. Analysis of mechanical parameters on the thromboembolism using a patient-specific computational model. *Biomech Model Mechanobiol* 2016;**15**:1295–305.

12 Neidlin M, Büsen M, Brockmann C, *et al.* A numerical framework to investigate hemodynamics during endovascular mechanical recanalization in acute stroke. *Int j numer method biomed eng* 2016;**32**:e02748.

13 Alastruey J, Parker KH, Peiró J, *et al.* Modelling the circle of Willis to assess the effects of anatomical variations and occlusions on cerebral flows. *J Biomech* 2007;**40**:1794–805.

14 Groen D, Richardson RA, Coy R, *et al.* Validation of patient-specific cerebral blood flow simulation using

For peer review only

transcranial doppler measurements. *Front Physiol* 2018;**9**:1–13.

15 Reymond P, Merenda F, Perren F, *et al.* Validation of a One-Dimensional Model of the Systemic Arterial Tree. *Am J Physiol - Hear Circ Physiol* 2009;**297**:208–22.

16 Liu B, Li Q, Wang J, *et al.* A Highly Similar Mathematical Model for Cerebral Blood Flow Velocity in Geriatric Patients with Suspected Cerebrovascular Disease. *Sci Rep* 2015;**5**:1–7.

17 Harst V Der, Joep J, J JW. Transcranial Doppler Versus CT- Angiography for Detection of Cerebral Vasospasm in Relation to Delayed Cerebral Ischemia After Aneurysmal Subarachnoid Hemorrhage A Prospective Single-Center Cohort Study The Transcranial doppler and CT-angiography for Inve. 2019;**44**:1–6.

18 Ghigo A, Wang X-F, Armentano R, *et al.* Linear and nonlinear viscoelastic arterial wall models: application on animals. *J Biomech Eng* 2017;**139**:011003.

19 Matthys KS, Alastruey J, Peiró J, *et al.* Pulse wave propagation in a model human arterial network: Assessment of 1-D numerical simulations against in vitro measurements. *J Biomech* 2007;**40**:3476–86.

FIGURE LEGENDS

Figure 1. Study flowchart

Figure 2. Schematic diagram of measuring point of diameter from CTA and flow velocity from TCD

Figure 3. SIMSCAPETM block diagrams of the arterial network with 33 segments, based on Alastruey et al [13]

For peer review only

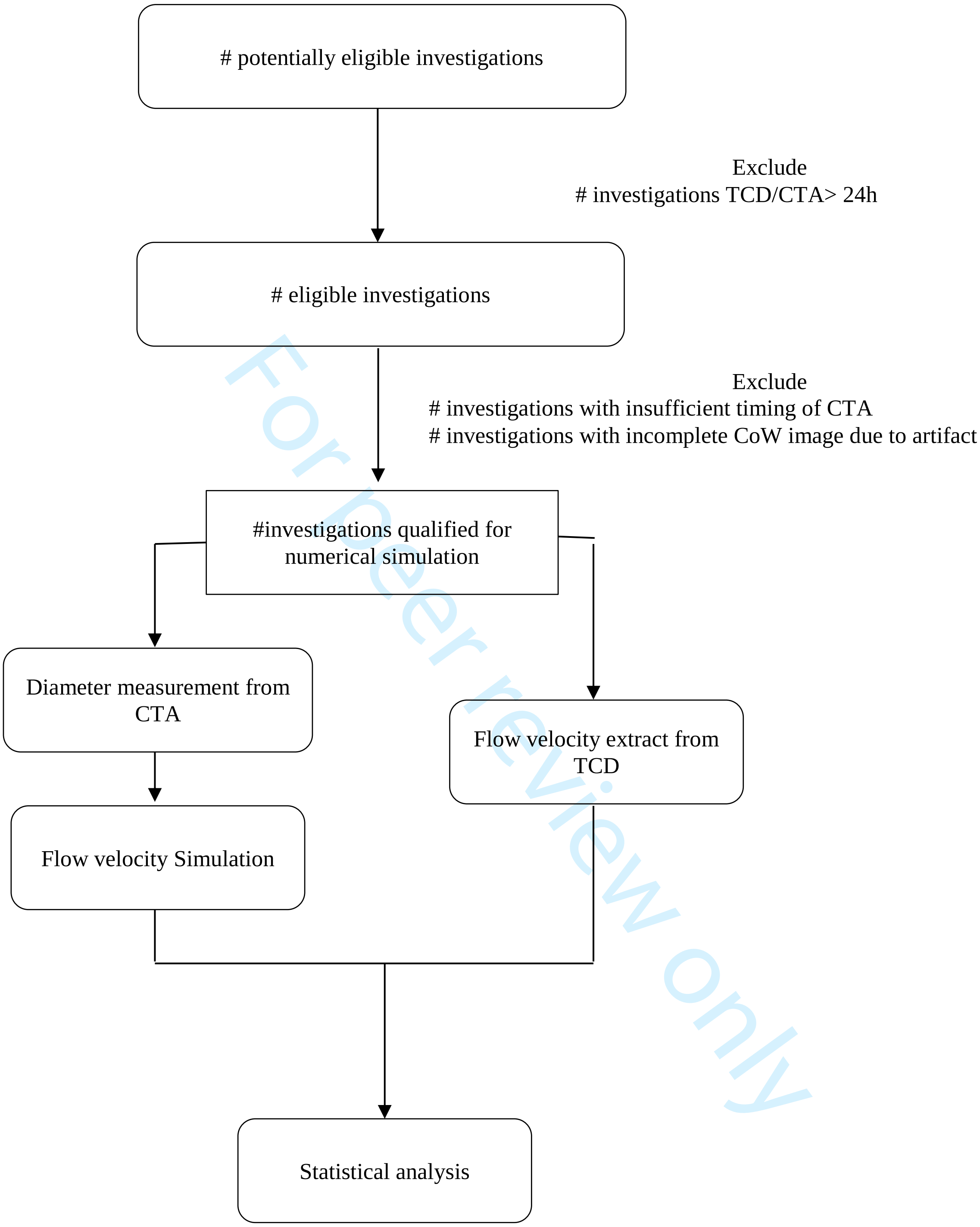

Figure 1. Study flowchart

For peer review only

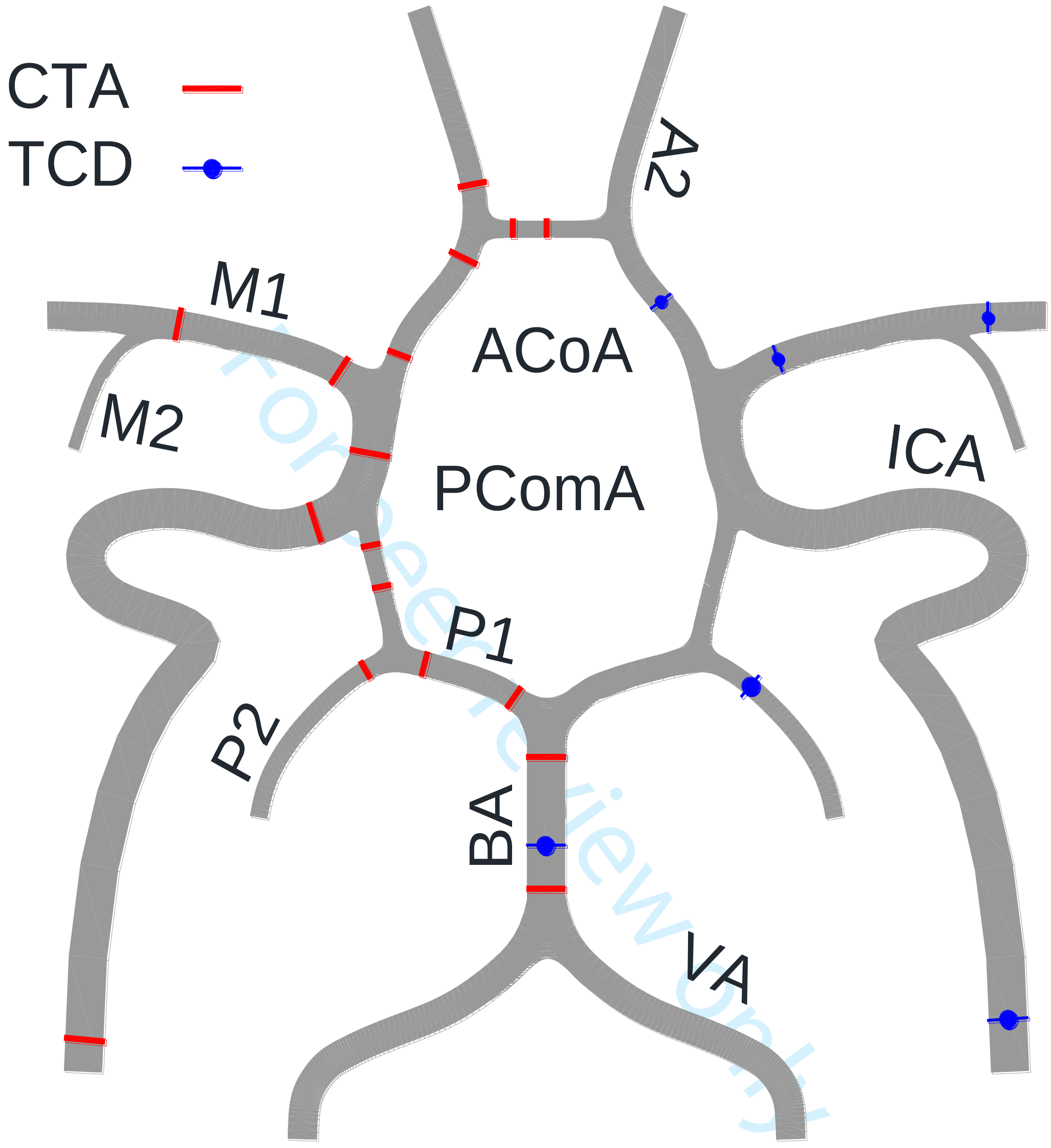

Figure 2. Schematic diagram of measuring point of diameter from CTA and flow velocity from TCD

For peer review only

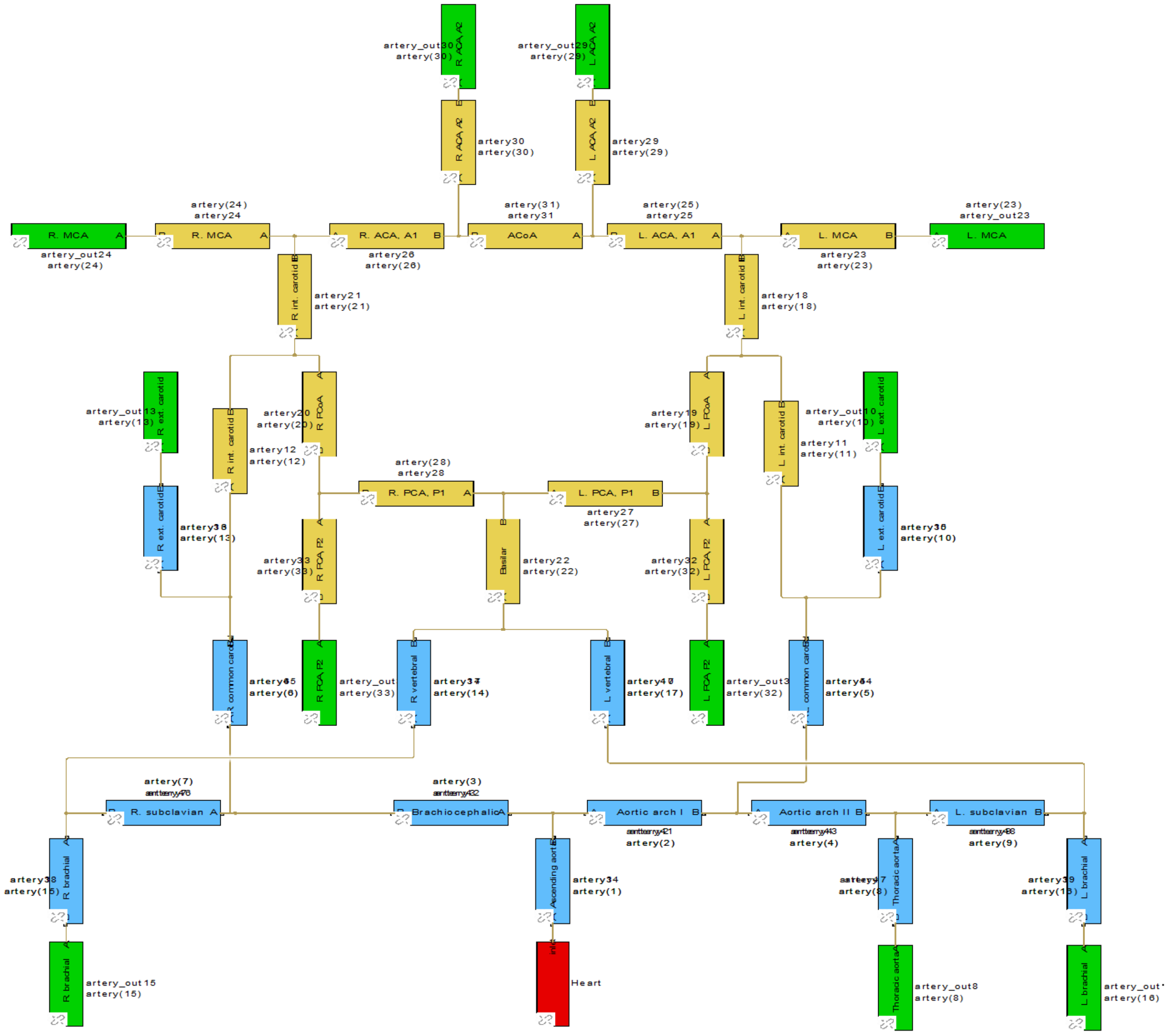

Figure 3. SIMSCAPETM block diagrams of the arterial network with 33 segments, based on Alastruey et al [13]
